# Stacked bilayer phosphorene: strain-induced quantum spin Hall state and optical measurement

**DOI:** 10.1038/srep13927

**Published:** 2015-09-15

**Authors:** Tian Zhang, Jia-He Lin, Yan-Mei Yu, Xiang-Rong Chen, Wu-Ming Liu

**Affiliations:** 1 Beijing National Laboratory for Condensed Matter Physics, Institute of Physics, Chinese Academy of Sciences, Beijing 100190, China; 2 Institute of Atomic and Molecular Physics, College of Physical Science and Technology, Key Laboratory of High Energy Density Physics and Technology of Ministry of Education, Sichuan University, Chengdu 610065, China

## Abstract

Bilayer phosphorene attracted considerable interest, giving a potential application in nanoelectronics owing to its natural bandgap and high carrier mobility. However, very little is known regarding the possible usefulness in spintronics as a quantum spin Hall (QSH) state of material characterized by a bulk energy gap and gapless spin-filtered edge states. Here, we report a strain-induced topological phase transition from normal to QSH state in bilayer phosphorene, accompanied by band-inversion that changes 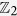 number from 0 to 1, which is highly dependent on interlayer stacking. When the bottom layer is shifted by 1/2 unit-cell along zigzag/armchair direction with respect to the top layer, the maximum topological bandgap 92.5 meV is sufficiently large to realize QSH effect even at room-temperature. An optical measurement of QSH effect is therefore suggested in view of the wide optical absorption spectrum extending to far infra-red, making bilayer phosphorene a promising candidate for opto-spintronic devices.

Recently, a new two-dimensional (2D) semiconductor material phosphorene has been exfoliated successfully^1^,^2^, and gained rapidly immense interests because of its high carrier mobility and large optical conductivity^3^,^4^,^5^,^6^,^7^,^8^,^9^,^10^,^11^,^12^,^13^. As compared with graphene and silicene^14^,^15^, hexagon lattice of phosphorene has higher buckling degree and stronger anisotropy, which indicates that the phosphorene is more sensitive to the strain manipulation^12^,^16^,^17^,^18^,^19^. As we known, strain is an effective way of tuning the electronic structure of the 2D semiconductor material, and even can create a nontrivial topology of occupied bands in a quantum spin Hall (QSH) state, accompanied by band inversion between occupied and unoccupied bands at the time reversal invariant momenta (TRIM) in the bulk Brillouin zone^20^,^21^,^22^,^23^. A semiconductor to metal transition for phosphorene has been predicted with a large strain^18^,^19^. However, due to large band gap, the required strain has exceeded the critical value that the stable phosphorene can exist. Bilayer phosphorene has a smaller bandgap than the monolayer phosphorene^24^,^25^, which offers more opportunity in tuning of indirect semiconductor, metal, and may even topological insulator. Furthermore, the weak interlayer van der Waals (*vdW*) interaction in the bilayer phosphorene allows for varieties of stacking orders^26^, which provides a new way to tune the electronic structure. Hence, the bilayer phosphorene should be a potential topological insulator. However, the topological phase transition in bilayer phosphorene has never been reported yet.

Quantum spin Hall state of matter has a charge excitation energy gap in the bulk and gapless spin-filtered edge-states on the boundary with a Dirac-cone-like linear energy dispersion^27^,^28^,^29^,^30^,^31^,^32^,^33^,^34^,^35^,^36^,^37^. The special edge-states, which are topologically protected by the time reversal symmetry, can survive the nonmagnetic scattering and geometry perturbations, thus open new ways for backscattering-free transport. Such systems have stimulated enormous research activities in condensed matter physics due to their novel quantum spin Hall effect and hence the potential application in quantum computation and spintronics^29^,^30^. An excellent QSH material should have a large bandgap and be easily fabricated. Graphene has a superior high carrier mobility but its bandgap is extremely small^31^,^32^,^33^, while bilayer bismuth (111) is potentially large bandgap material but its fabrication is usually difficult due to its strong interlayer bonding^34^,^35^. Bilayer phosphorene has not only a high carrier mobility and a large bandgap but also easy fabrication. Hence, bilayer phosphorene can be a potential excellent candidate for future applications in QSH devices, such as topological field-effect transistors and spin valve devices.

In this work, we will investigate a quantum spin Hall state of bilayer phosphorene by tunning strain and stacked order, then find a topological phase transition from normal to QSH state accompanied by a band inversion that changes 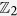 number from 0 to 1. Furthermore, the direct-indirect bandgap and semiconductor-metal transitions can be observed by adjusting the weight of *vdW* interaction. Our results reveal that the tuning of topological behavior in bilayer phosphorene is dependent on interlayer stacking order and the direction of the applied in-plane strain that should be along either armchair or zigzag direction. When the bottom layer is shifted by 1/2 unit cell along the zigzag or the armchair direction with respect to the top layer, the maximum topological bandgap can reach up to 92.5 meV, which is sufficiently large to realize the quantum spin Hall effect even at room temperature. The optical property of bilayer phosphorene is examined based on a real-space and real-time time-dependent density functional theory, which shows that the optical absorption spectrum of bilayer phosphorene becomes wide and is even extended to far-infra-red region in QSH state. Such improvement in optical response is indispensable for the broadband photodetection. An optical experimental setup is therefore improved to measure the QSH effect in bilayer phosphorene.

## Results

### Crystal structures and electronic structures of bilayer phosphorene for four stacking orders.

**Figure 1:**
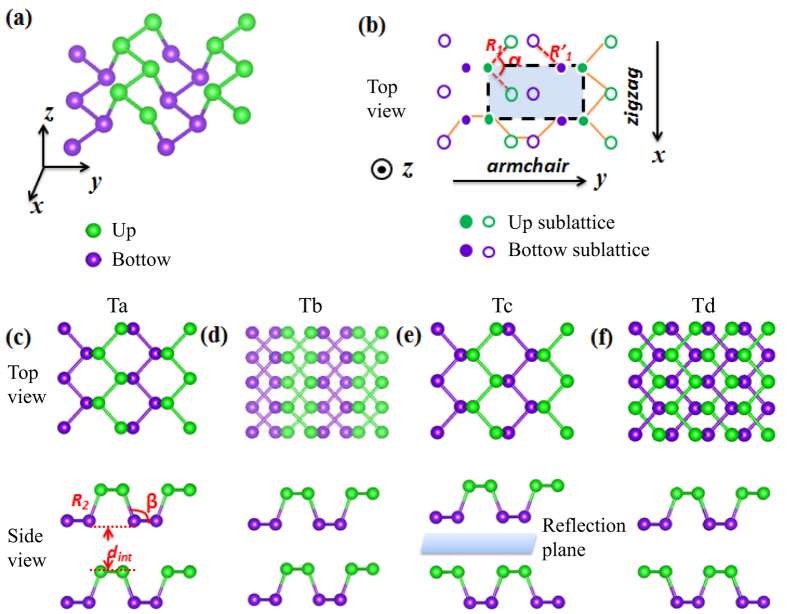
Crystal structures of bilayer phosphorene for four stacking orders. (**a**) The monolayer black phosphorus (phosphorene), where the top and bottom P sublattices in a nonplanar monolayer are represented by green and purple balls. (**b**) The projection of monolayer black phosphorus on *x*-*y* plane, where filled and opened circles are two different sublattices, *R*_1_ and 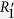 are P-P bond lengths in up and bottom sublattices, and *α* is the angle between two *R*_1_ bonds. One unit cell has four atoms, as denoted by the blue shadow. (**c**–**f**) The top and side views of the four different stacking orders: (Ta) the top layer is stacked vertically on the bottom layer, (Tb) the bottom layer is shifted by half of one unit cell along x or y with respect to the top layer, (Tc) the bottom layer is shifted by one unit cell along x or y direction with respect to the top layer, and thus the top and bottom layers are mirror images of each other, and (Td) the bottom layer is shifted by one and a half of one unit cell along x or y direction. Here, *R*_2_ is the P-P bond length between up and bottom sublattices of a nonplanar monolayer, *β* is the angle between the *R*_2_ and 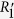 bonds, and *d*_*int*_ is the smallest interlayer distance.

The crystal structure of phosphorene is given in Fig. 1. Bilayer phosphorene can be viewed as cleaved from the (0001) surface of black phosphorus (space group *Cmca*), which allows various stacking orders due to its weak interlayer van der Waals (*vdW*) interaction, as shown in Fig. 1(a,b). We consider four possible high-symmetry stacking orders: (Ta) the top layer is stacked vertically on the bottom layer (space group *Pmna*), (Tb) the bottom layer is shifted by 1/2 unit cell along *x* (zigzag) or *y* (armchair) direction with respect to the top layer (space group *Pbcm*), (Tc) the bottom layer is shifted by one unit cell along *x* or *y* direction with respect to the top layer, and thus the top and bottom layers are mirror images of each other (space group *Pmma*), and (Td) the bottom layer is shifted by 3/2 unit cells along *x* or *y* direction with respect to the top layer (space group *Pccm*), as shown in Fig. 1(c–f). Table 1 gives the optimized lattice constants, the bond lengths and the other structural parameters of the bilayer phosphorene for the four stacking orders, which is well consistent with the previously theoretical data^26^ with errors lower than 0.5%. For the different stacking orders, the bond length *R*_1_ and 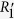 are almost same, being shorter than the bond length *R*_2_. The bond angles *α* is smaller than *β*. The smallest interlayer distance *d*_*int*_ is 3.503 Å (Ta), 3.085 Å (Tb), 3.739 Å (Tc) and 3.291 Å (Td). Among the four stacking orders, the Tb stacking order has the lowest cohesive energy. Therefore, the phonon spectrum of the Tb stacking order is further calculated, which shows the excellent structural stability (see Supplementary information). The cohesive energies of the Ta, Tc, Td stacking orders are slight larger than the Tb stacking order with the difference less than 10 meV, which indicates that the Ta, Tc and Td stacking orders also have the excellent structural stability.

**Figure 2:**
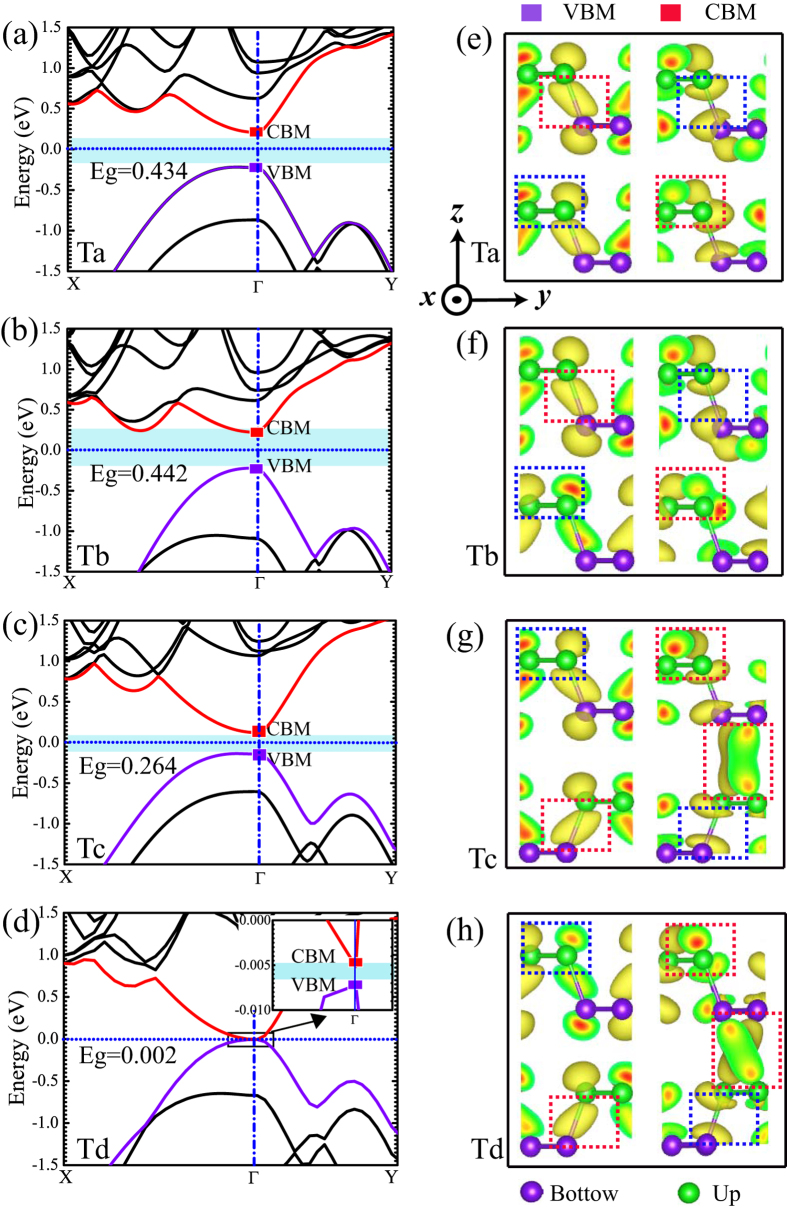
Electronic structures of bilayer phosphorene for four stacking orders by PBE method. (**a**–**d**) The band structures. The valence band and the conduction band are represented by purple and red lines with the valence band maximum (VBM) and the conduction band minimum (CBM) being denoted by purple and red squares. The bandgap *E*_*g*_ is highlighted by blue shadow. The inset in (**d**) shows the region close to the Fermi level. The Fermi level is set to zero, and Γ (0.0, 0.0, 0.0), X (0.0, 0.5, 0.0) and Y (0.5, 0.0, 0.0) refer to special points in the first Brillouin zone. (**e**–**h**) The isosurface of band-decomposed charge densities on the *y*−*z* plane of VBM and CBM, where top and bottom P sublattices in a nonplanar monolayer are represented by green and purple balls. A bond (antibond) is circled by red (blue) box. The isosurface is set to be 0.0037 e/Å^3^.

The electronic structure of the bilayer phosphorene is given in Fig. 2. Shown in Fig. 2(a–d) are the band structures of the four different stacking orders obtained by using Perdew-Burk-Ernzerhof (PBE) functional^38^, which gives the bandgap *E*_*g*_ = 0.434 eV (Ta), 0.442 eV (Tb), 0.264 eV (Tc) and 0.002 eV (Td) at Γ point. Since our hybrid Heyd-Scuseria-Emzerhof (HSE06)^39^,^40^ calculations indicate that the PBE *E*_*g*_ values are underestimated about 0.56 eV, the *E*_*g*_ of the bilayer phosphorene should be larger than 0.56 eV for the four different stacking orders, which is well consistent with the previous HSE06 data^26^. The iso-surfaces of the band-decomposed charge density of the valence band maximum (VBM) and the conduction band minimum (CBM) at Γ point show that the four stacking orders have the similar charge density distribution and bonding character, as shown in Fig. 2(e–h). Basically, the charge density distributions of the VBM and CBM in one nonplanar monolayer have no effect on the stacking order: an anti-bonding-like feature in one sublayer and a bonding-like feature between two sublayers are visible for the VBM, while the bonding-like features in one sublayer and the anti-bonding-like features between two sublayers are found for the CBM. On the contrary, the charge density distributions of the VBM and CBM between two layers are dependent on the stacking order: the sign of the chemical bond between two layers is not visible for the VBM in the four stacking orders, but a chemical bonding-like character between two layers for the CBM is found in the Tc and Td stacking orders, which is absent in Ta and Tb stacking orders. The bonding features for the CBM in the Tc and Td stacking orders push their CBM down to the low energies, which leads to the smaller bandgaps than the Ta and Tb stacking orders. Therefore, it is confirmed that the different chemical bonding in the interfacial area between two layers is related to the bandgap *E*_*g*_ for the four stacking orders.

### Quantum spin Hall state induced by in-plane strain in bilayer phosphorene

**Figure 3:**
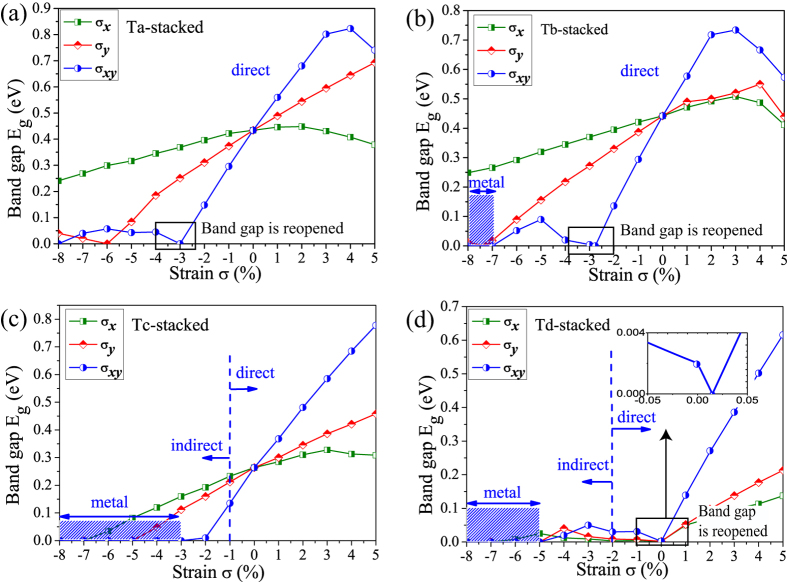
Bandgap *E*_*g*_ with in-plane strain *σ* of bilayer phosphorene for four stacking orders by PBE method. Three types of strains *σ*_*x*_, *σ*_*y*_, and *σ*_*xy*_ are considered, which are along x (zigzag), y (armchair) and both x and y directions, respectively. The positive (negative) *σ* values corresponds to the tension (compression) strain. There exists a critical compression stain for the four stacking orders, where the closed bandgap turns to be reopened, corresponding to a band inversion process, as denoted by black box in (**a**–**d**). For the Tc and Td stacking orders, there occurs a direct-indirect bandgap transition up to a critical compression strain, as denoted by blue dash line in (**c**) and (**d**). For the Tb, Tc, and Td stacking orders, the bilayer phosphorene becomes the metal state, as the compression strain increases further, as denoted by blue shadow.

Figure 3 illustrates the variation of the bandgap of the bilayer phosphorene versus the in-plane strain for the four stacking orders, where *σ*_*x*_, *σ*_*y*_ and *σ*_*xy*_ are the in-plane strains along x-direction, y-direction, and both x- and y- directions, respectively. When the in-plane strain is applied, the lattice constants along the other directions are relaxed fully until the residual force on each atom is less than 0.01 eV/Å in order to ensure the complete relaxation of the crystal structure for the strained bilayer phosphorene. In our work, the in-plane uni-axial strain is defined as 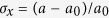 and 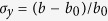, where *a*,*b* are lattice constants along the *x*,*y* directions under strain, respectively, and *a*_0_,*b*_0_ are the corresponding equilibrium lattice constants without strain. The in-plane strain *σ*_*xy*_ is loaded synchronously in the x- and y-directions. In experiment, the in-plane strain on the bilayer phosphorene can be realized by bending its flexure substrate similar to graphene, where the amount of the strain is proportional to 2D mode position of the bilayer phosphorene^41^. Bandgaps of the four stacking orders are more sensitive to *σ*_*xy*_, as compared with *σ*_*x*_, *σ*_*y*_. When *σ*_*xy*_ > 0 (tensile), the bandgap increases with *σ*_*xy*_, then turns to decrease at a critical value, being 4.0% (Ta), 3.0% (Tb), 5.0% (Tc and Td). When *σ*_*xy*_ < 0 (compression), the bandgap decreases with *σ*_*xy*_ and becomes zero at *σ*_*xy*_ = −3.0% (Ta and Tc), −2.77% (Tb) and 0.02% (Td). However, to our surprised, after the bandgaps are closed, *E*_*g*_ turns to increase again for the Ta, Tb, and Td stacking orders [see the inset Fig. 3(d)]. Such close-reopen process of the bandgap indicates a possible topological phase transition. For Tc stacking order, the reopening of the bandgap is not seen up to *σ*_*xy*_ = −8.0%, which rules out the possibility of topological phase transition. Then the bandgap turns to decrease again with *σ*_*xy*_ until the bilayer phosphorene becomes to be the metal state at *σ*_*xy*_ = −8.0% (Ta), *σ*_*xy*_ = −7.0% (Tb), *σ*_*xy*_ = −3.0% (Tc), and *σ*_*xy*_ = −5.0% (Td) finally. Besides, we also find a direct-indirect bandgap transition when *σ*_*xy*_ = −1.0% (Tc) and −2.0% (Td).

**Figure 4:**
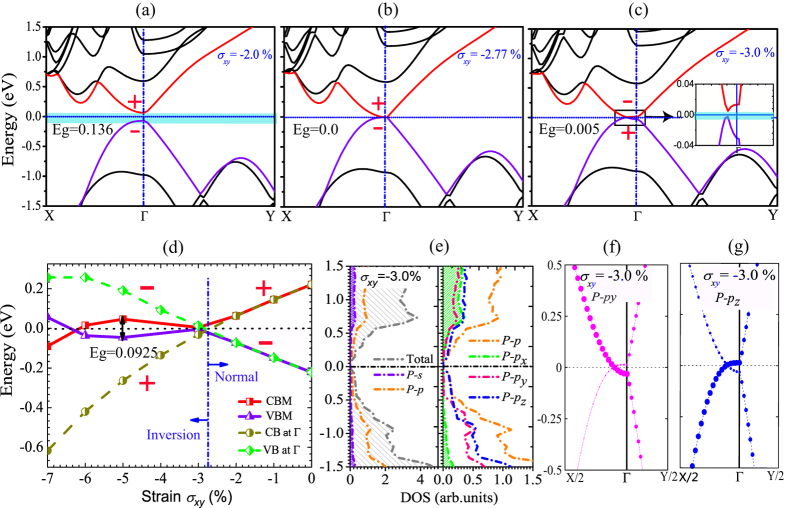
Electronic structures of Tb-stacked bilayer phosphorene by PBE method. (**a**–**c**) The band structures when the in-plane strain *σ*_*xy*_ is −2.0%, −2.77% and −3.0%, where the valence band (VB) and conduction band (CB) are represented by the purple and red lines. The inset in (**c**) shows the region close to the Fermi level. The Fermi level is set to zero, and Γ (0.0, 0.0, 0.0), X (0.0, 0.5, 0.0) and Y (0.5, 0.0, 0.0) refer to special points in the first Brillouin zone. (**d**) The evolution of the VB and the CB at Γ with the compression strain *σ*_*xy*_. At Γ point under *σ*_*xy*_ = 0, the CB and the VB have even (‘+’) and odd (‘−’) parities, respectively. Their parities are exchanged when the compression *σ*_*xy*_ < −2.77%, which indicates a clearly band inversion. The maximum bandgap *E*_*g*_ after the band inversion is obtained about 92.5 meV, where the maximum of the VB and the minimum of the CB are not located at the Γ point. (**e**) The density of states of the Tb-stacked bilayer phosphorene when *σ*_*xy*_ = −3.0%, where the total density of states (DOS) is represented by the gray dotted lines, the *s*- and *p*-orbitals of P atom are represented by the purple and orange dotted lines, and the *p*_*x*_, *p*_*y*_ and *p*_*z*_ orbitals are represented by green, red and blue dotted lines. (**f**) and (**g**) The *p*_*y*_ and *p*_*z*_ orbital-projected band structures of Tb-stacked bilayer phosphorene, where the radii of circles are proportional to the weight of corresponding orbital.

Next, the electronic structure of the Tb-stacked bilayer phosphorene is given in Fig. 4. At Γ point under *σ*_*xy*_ = 0, the conduction band (CB) and the valence band (VB) have the even (‘+’) and odd (‘−’) parities, respectively. Shown in Fig. 4(a–c) are the electronic band structures for the Tb stacking order under different *σ*_*xy*_ values. The CB and VB at Γ point tend to approach together as *σ*_*xy*_ increases, then become overlapped under *σ*_*xy*_ = −2.77%. For larger compression strain (*σ*_*xy*_ = −3.0%), the CB and VB at Γ point are separated because of the repulsion between them. Most remarkably, the parities of the CB and VB at Γ are exchanged when the compression strain *σ*_*xy*_ < −2.77%, as shown in Fig. 4(d), which shows the band inversion of the VB and CB at Γ. Such band-inversion character is also observed in the density of states (DOS) and the orbital-projected band structures when *σ*_*xy*_ = −3.0% [see Fig. 4(e–g)]. The *p* orbital makes a significant contribution to the total DOS. The conduction band near *E*_*F*_ mainly comes from the *p*_*y*,*z*_ obitals, but the bottom of the conductor band originates mostly from the *p*_*z*_. The valence band near *E*_*F*_ mainly comes from the *p*_*z*_ obital, but the top of the valence band originates mostly from the *p*_*y*_ orbital, which indicates an band inversion process when the compression strain *σ*_*xy*_ is increased.

Further, we calculate the 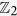 number of the Tb-stacked bilayer phosphorene when *σ*_*xy*_ = 0 and −3.0%, by using the method of Fu and Kane^42^. Such method is valid since the Tb stacking order has both spatial invention and time reversal symmetries (four time reversal invariant points in the 2D Brillouin zone). Inversion center in the crystal ensures 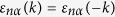, where 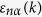 is the electron energy for the *n*-th band with spin index *α* at k wave vector in the Brillouin zone. The time reversal symmetry makes 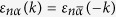, where 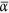 is the spin opposite to *α*. The calculated parities of all occupied bands at four time-reversal invariant momenta are summarized in Table 2. We can find that the product of parities of occupied bands contributes to a +1 parity at the four time-reversal invariant momenta when *σ*_*xy*_ = 0, yielding a trivial topological invariant 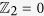. As the strain is increased up to *σ*_*xy*_ = −3.0%, the product of parities of occupied bands is −1 at Γ but +1 at the three other time-reversal invariant momenta like X, Y, and S. The Tb-stacked bilayer phosphorene under *σ*_*xy*_ = −3.0% is identified as topological insulators with 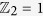. The results shown in Fig. 4(d) suggest that the Tb-stacked bilayer phosphorene maintain to be the QSH state under the compression strain *σ*_*xy*_ = −2.77 ~ −7.0%, where the maximum topological bandgap *E*_*g*_ = 92.5 meV is obtained when *σ*_*xy*_ = −5.0%.

**Figure 5:**
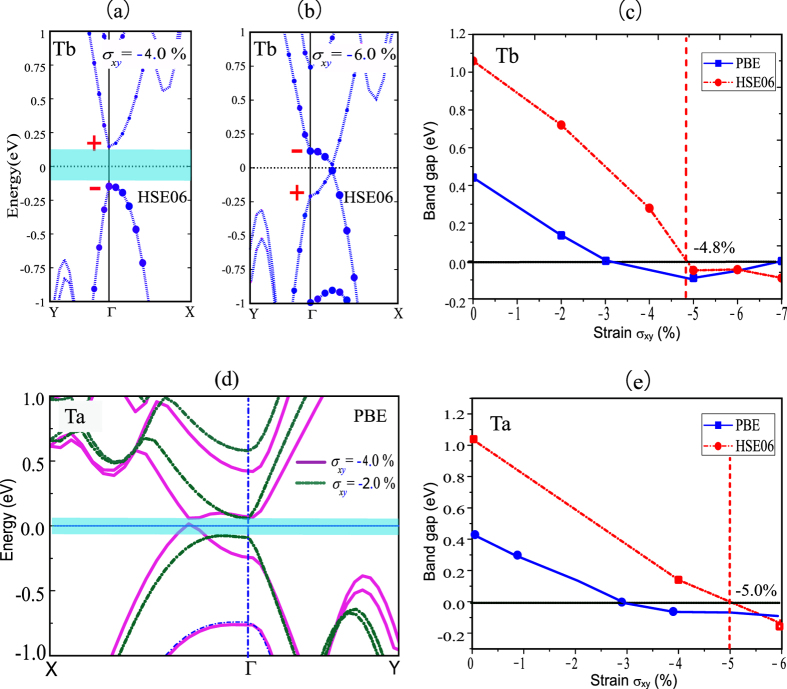
Electronic structures of bilayer phosphorene for Ta and Tb stacking orders by PBE and HSE06 methods. (**a**) and (**b**) The orbital-projected band structures of the Tb-stacked bilayer phosphorene by the HSE06 method when *σ*_*xy*_ = –4.0% and −6.0%, respectively, where the weight of P-*p*_*z*_ is represented by the radii of blue circles. The even parity denoted by + , and the odd parity denoted by −. (**c**) The bandgaps for the Tb stacking order by the PBE and HSE06 methods change with *σ*_*xy*_. (**d**) The band structures of the Ta-stacked bilayer phosphorene by the PBE method when the in-plane strain *σ*_*xy*_ is −2.0% and −4.0%. (**e**) The bandgaps for the Ta stacking order by the PBE and HSE06 methods change with *σ*_*xy*_. The obtained bandgaps by the PBE and HSE06 methods are represented by blue line and red dotted line, respectively. The Fermi level is set to zero, and Γ(0.0, 0.0, 0.0), X(0.0, 0.5, 0.0) and Y(0.5, 0.0, 0.0) refer to special points in the first Brillouin zone.

The PBE method may underestimate the bandgap compared with reality, which leads to a low evaluation of the critical strain needed to produce a quantum spin Hall state. In order to avoid such problem, we have carried out the band structure calculations of the possible topological insulators (Ta and Tb stacking orders) under the in-plane strains by the HSE06 method which has been proven reliable for few layer phosphorene systems^26^. Fig. 5(a,b) present the P-*p*_*z*_ orbital-projected band structures of the Tb-stacked bilayer phosphorene by the HSE06 method when *σ*_*xy*_ = −4.0% and −6.0%, respectively. We can find that the top of the valence band and the bottom of the conduction band for the Tb stacking order exchange the weight of P-*p*_*z*_ orbital when the strain *σ*_*xy*_ = −6.0%, which shows a band inversion. Actually, the Tb-stacked bilayer phosphorene has already been in quantum spin Hall state when the strain is beyond −4.8%, as shown in Fig. 5(c). The corresponding strain range where the Tb-stacked bilayer phosphorene maintain to be the QSH state is −4.8 ~ −10.0% by the HSE06 method. It is worth noting that the lattice structure of the Tb-stacked bilayer phosphorene is still stable under the compression strain up to −5.0% by calculating its phonon spectrum (see Supplementary information), which is critical for the practical applications. The topological phase transition for the Ta stacking order induced by in-plane strain is proven by the same method. Like the Tb stacking order, the Ta stacking order also undergoes the bandgap closed and reopened process with the increasing *σ*_*xy*_, as shown in Fig. 5(d), which shows a band inversion. Shown in Fig. 5(e) is the evolution of the bandgap with *σ*_*xy*_, which indicates that the PBE bandgap is underestimated compared with the HSE06 bandgap. Hence the critical strain *σ*_*xy*_ (about −5.0%) obtained by the HSE06 method, where the Ta-stacked bilayer phosphorene is in quantum spin Hall state, is higher than that (about −3.0%) by the PBE method. For the Td stacking order, the VBM and CBM have the same parity, which rules out the possibility of topological phase transition.

### Semiconductor-metal transition induced by interlayer interaction in bilayer phosphorene

**Figure 6:**
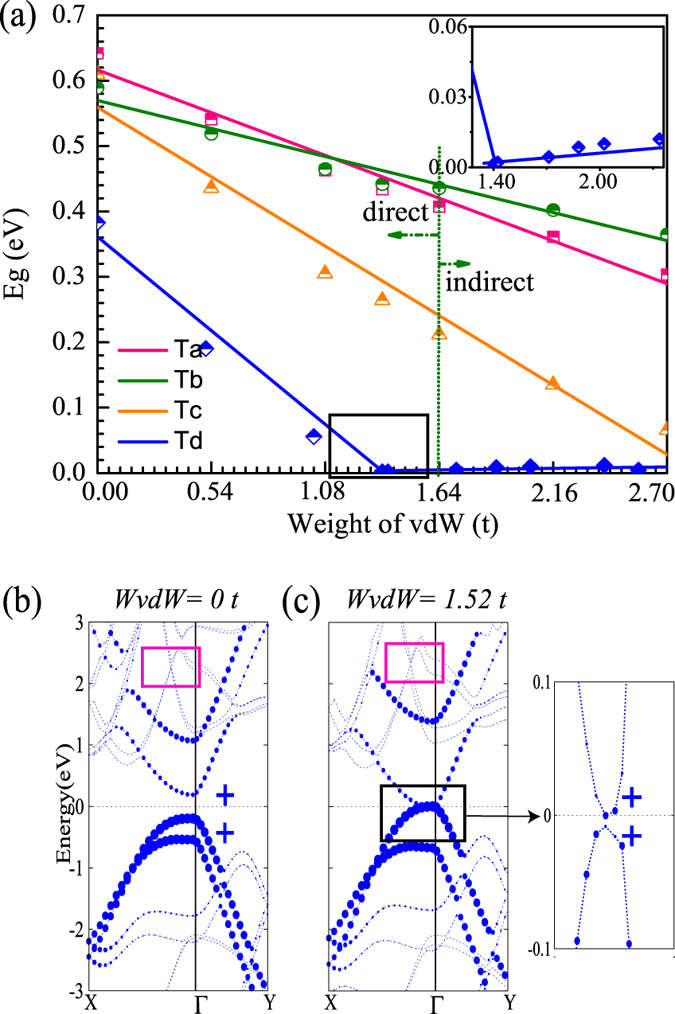
Electronic structures of bilayer phosphorene for four stacking orders by PBE method under different *WvdW* values. (**a**) The bandgap *E*_*g*_ changes with *WvdW* interaction. We find the bandgap of the Td-stacked bilayer phosphorene is reopened when *WvdW* interaction is up to a critical value, as shown in the inset. (**b**) and (**c**) The orbital-projected band structures of the Td-stacked bilayer phosphorene when *WvdW* = 0 *t* and 1.52 *t*, where the *p*_*z*_ component is denoted by circle, and the weight of *p*_*z*_ orbital is proportional to the radius of the circle with the even parity denoted by + . The doubly degenerate of the energy band in partial zone (the pink box) is broken with the increasing *WvdW* interaction. The Fermi level is set to zero, and Γ(0.0, 0.0, 0.0), X(0.0, 0.5, 0.0) and Y(0.5, 0.0, 0.0) refer to special points in the first Brillouin zone.

The electronic band structure as function of the weight of van der Waals interaction between the top and bottom layers of the bilayer phosphorene is shown in Fig. 6. The weight of van der Waals interaction is incorporated into our DFT calculations by using a semi-empirical van der Waals approach, known as DFT-D2 method^43^. The total energy is defined as *E*_*DFT−D*_ = *E*_*KS−FGT*_ + *W E*_*vdW*_, where *E*_*KS−DFT*_ is the Kohn-Sham DFT total energy, *E*_*vdW*_ is the *vdw* energy, and *W* is a scaling factor in order to consider the weight of van der Waals (*WvdW*) interaction.

The variation of the bandgap *E*_*g*_ of the bilayer phosphorene versus the *WvdW* interaction is illustrated in Fig. 6(a). The *E*_*g*_ tends to decrease with the increasing *WvdW* interaction for all four stacking orders. When the increasing *WvdW* interaction is beyond 2.70 *t* (*t* is the real value for the *WvdW* interaction), *E*_*g*_ for each stacking order will decrease gradually to zero, which indicates that the VBM and CBM of the bilayer phosphorene have been overlapped, yielding a semiconductor to metal transition. For the Td stacking order, the decrease of *E*_*g*_ versus *WwdW* is much stronger than the Ta, Tb, and Tc stacking orders. Very specifically, we find a bandgap closed and reopened process for the Td stacking order, as shown in the inset of the Fig. 6(a), whereas this phenomenon is absent for the Ta, Tb and Tc stacking orders. The P-*p*_*z*_ orbital-projected band structures of the Td-stacked bilayer phosphorene are presented in Fig. 6(b) under *WvdW* = 0 *t* and Fig. 6(c) under *WvdW* = 1.52 *t*. We find that the energy band is doubly degenerate in partial zone [see the pink box in Fig. 6(b)] under *WvdW* = 0 *t*. However, as the *WvdW* interaction increases up to *WvdW* = 1.52 *t*, this band degeneracy is broken [see the pink box in Fig. 6(c)], which can be due to the reason that the top and the bottom layers of the Td-stacked bilayer phosphorene tend to be bonded as companied with decrease of *d*_*int*_. Furthermore, as accompanied by the bandgap closed and reopened process, the VBM and CBM for the Td stacking order exchange the weight of P-*p*_*z*_ orbital, indicating the band inversion of the VBM and CBM. However, as mentioned above, the VBM and CBM for the Td stacking order have the same parity, which rules out the possibility of topological phase transition. Hence, despite that the *WvdW* interaction causes the decrease of *E*_*g*_ and the break of the band degeneracy for the four stacking orders, but does not cause the topological phase transition, as shown in our computations.

Our calculations establish the basic conditions for materials to exhibit strain-induced topological phase transition. First, the direction of the applied strain on materials should be along the bonding direction, i.e., the direction with the maximum atomic wave function overlap. For the bilayer phosphorene, this direction should be along the armchair or zigzag direction [see Fig. 2(e–h) above]. Second, the VBM and CBM of material should have the different parities, which is not the details of the atomic orbitals or bond types but the different signs of overlap integrals of the atomic orbitals. Hence, the topological phase transition of the Td-stacked bilayer phosphorene induced by strain is impossible. Third, materials should have direct bandgaps located at the time reversal invariant momenta before the transition. If not, the bandgaps would make the metal phase occur before band inversion, just like in the case of the Tc-stacked bilayer phosphorene.

### Optical response of bilayer phosphorene and optical measurement of quantum spin Hall effect

**Figure 7:**
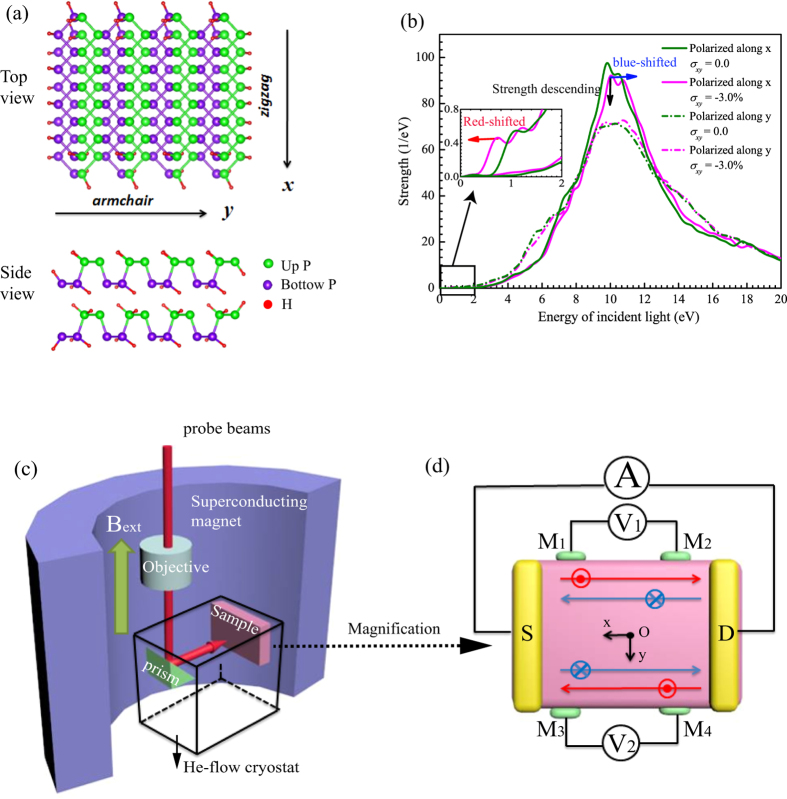
Optical respond of Tb-stacked bilayer phosphorene and optical measurement of quantum spin Hall effect. (**a**) The top and side views of the crystal structure, where the hydrogen atoms (red balls) is used to passivate the dangling *σ* bonds on the boundary. (**b**) The optical absorption spectra when the compression strain *σ*_*xy*_ = 0.0 and −3.0%. Two polarized directions are considered, which are along x (zigzag) and y (armchair) directions. Red- (blue-) shifted of the absorption peaks is highlighted by red (blue) arrow, and the strength descending of the absorption peak is highlighted by black arrow. (**c**) Schematic of the experimental geometry to detect the quantum spin Hall effect. We place the sample in a He-flow cryostat and keep it at 30 K. The probe beam is incident upon the sample through a objective and a prism mirror placed in the He-flow cryostat. The objective is used to ensure the beam to focus on a controlled circular spot, and the prism mirror is used to bent the probe beam to 90°. All the equipments are placed in a superconductor magnet that can induce a magnetic field *B*_*ext*_. (**d**) Magnification of the sample configure in (**c**), where the source and drain electrodes are denoted as ‘S’ and ‘D’, four terminals are represented by M_1_, M_2_, M_3_ and M_4_, and the spin-up and spin-down states are represented by red and blue lines. The center of the sample (“O” point) is set as the origin of the coordinate. The ac electronic current between the source and the drain electrodes can be measured by the ammeter A, and the electronic voltage between the terminal M_1_ (M_3_) and M_2_ (M_4_) can be measured by the voltmeter V_1_ (V_2_).

As the most primary structure inheriting from the bulk black phosphorus, the Tb stacking order has the highest structural stability and is most facile to be fabricated in experiment among the four stacking orders. Therefore, the Tb-stacked bilayer phosphorus would be the best phosphorus candidate for the future optical electronic devices. The optical response property of the Tb-stacked bilayer phosphorene is investigated, as shown in Fig. 7. The optical response calculation is conducted for 4 × 4 supercell of Tb-stacked bilayer phosphorene [see Fig. 7(a)]. In Fig. 7(b), we compare the optical response of the Tb-stacked bilayer phosphorene in the normal state (*σ*_*xy*_ = 0) and the QSH state (*σ*_*xy*_ = −3.0%), where the structural stability in QSH state is proved by calculating the phonon spectrum (see Supplementary information). Our results show that the photonic band-gap is decreased in the QSH state greatly, for example, the obtained photonic band-gap (PBG) is 0.5 eV in the normal state but only 0.01 eV in the QSH state for the case of the incident light polarized along the armchair direction. There exists two adsorption peaks in the optical adsorption spectrum. The first adsorption peak appears in the region that the optical strength of the incident light is less than 2 eV and the corresponding adsorption strength is also very low. When the incident light is polarized along the armchair direction, the first adsorption peak shows a red-shift in the QSH state [see red arrow in inset Fig. 7(b)], which leads to wider absorption zone that is highly desirable in broadband photodetection. The second adsorption peak occurs at the region of the optical strength of the incident light around 10 eV, which indicates that the bilayer phosphorene occurs a *σ* plasmon resonance. When the incident light is polarized along the zigzag direction, the second absorption peak becomes smaller and shows a blue shift in the QSH state, as compared with the normal state [see the black and blue arrows in Fig. 7(b)]. The blue shift is because that the increased interatomic spacing in the zigzag direction causes the larger resonance level spacing. The mechanism of the optical absorption in low-energy resonance behind the resonance phenomena is further analyzed in terms of the induced charge response in real-time propagation. The induced electron and hole charge densities tends to be separated and finally located at two edges. The induced charge density in the QSH state is larger than that in the normal state, emerging in the center areas in addition to the two edges. This is attributed to that the shielding effect becomes stronger in the QSH state when the interatomic spacing becomes smaller due to the in-plane strain (see Supplementary information).

We propose an experimental setup [see Fig. 7(c,d)] to measure QSH effect of strained Tb-stacked bilayer phosphorene by using Scanning Kerr rotation microscopy. Our setup has the similar working principle with the previous optical measurement of local spin accumulation at the sample edges in n-GaAs and n-InGaAs^44^,^45^,^46^, but combines with the electronic measurement. Experimentally, because the transverse spin currents do not lead to net charge imbalance across the sample in nonmagnetic systems, the spin Hall effect is difficult to be observed by the simple electrical measurement. Hence, we employ the Scanning Kerr rotation microscopy to image the spin polarization at the edges of the samples that has 320-*μ*m-long and 100-*μ*m-wide, as shown in Fig. 7(c). The probe beam is bent 90° by a prism mirror placed in the He-flow cryostat, then is incident upon the samples through an objective lens with 0.73 *μ*m lateral spatial resolution, which ensures that the beam is focusing on a circular spot with a full width at half-maximum of 1.1 mm^44^. An image from the Scanning Kerr rotation microscopy is taken by moving the cryostat integrated on a piezo nanoscanning stage, just like in the case of GaAs^46^. The Tb-stacked bilayer phosphorene is grown epitaxially on a substrate of comparable thickness, which makes the layers strained due to a mismatch in lattice parameter between the film and substrate materials. We set the center of the sample (“O” point) as the origin of the coordinate, and *x* and *y* axes are taken to be across and along the sample channel, respectively. This channel is defined by the standard photolithography and the wet etching. Kerr rotation is measured as a function of magnetic field (*B*_*ext*_) and position (*x*, *y*), which is at a maximum at the two the channel edges when *B*_*ext*_ = 0 mT and falls off rapidly with distance from the edge^44^. This indicates an accumulation of electron spins polarized at the edges, which is also a strong signature of the spin Hall effect because the spin polarization is out-of plane and changes sign for opposing edges^47^,^48^,^49^. Secondly, the two-terminal conductance *G*_*SD*_ can be measured by using the electronic measurement, as shown in Fig. 7(d). When sample is a normal insulator, *G*_*SD*_ should vanish at low temperatures, whereas *G*_*SD*_ should equal a value ~*ne*^2^/*h* (*n* is the integer, *e* is the electronic charge and *h* is the Planck constant). It is worth noting that the electronic voltage V_1_ and V_2_ vanish between M_1_ and M_2_ and between M_3_ and M_4_, which is one of the remarkable manifestations of the QSH state^50^.

## Discussion

We have proven that the Ta- and Tb-stacked bilayer phosphorene are excellent topological materials when the compression strain loaded synchronously in the x- and y-directions is larger than about 3%. The quantum spin Hall (QSH) state of the bilayer phosphorene is revealed due to the band inversion between the valence band maximum of *p*_*z*_ orbital and the conduction band maximum of *p*_*y*_ and *p*_*z*_ orbitals. Our results show that the strain manipulation is very effective for the bilayer phosphorene. In addition to the semiconductor-to-metal transition, strain can also induce the nontrivial topological band structure of the bilayer phosphorene. The obtained maximum topological bandgap is high up to 92.5 meV in the Tb-stacked bilayer phosphorene, implying that the QSH effect of bilayer phosphorene is observable even at room temperature. On the other hand, as compared with the chemical decoration and the electric field, the strain manipulation is more feasible in experiment, considering the current microfabrication technique, for example, heterepitaxy of the bilayer phosphorene grown on substrates of smaller lattices. Our results confirm that the bilayer phosphorene is an ideal material to study novel quantum states, showing new potential for future applications in spintronics devices such as topological field-effect transistors and spin valve devices.

Furthermore, the strain is also an excellent way to tuning optical property besides those aforementioned. The optical absorption spectrum of the strained bilayer phosphorene becomes broadened, and is even extended to the far-infra-red region and leads to a wider range of brightness. An improvement optical experimental equipment is designed therefore to measure the QSH effect in strained bilayer phosphorene, which shows that the strain-induced topological bilayer phosphorene can also be used as an outstanding opto-spintronic device.

## Methods

### 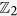 calculation

We use the method of Fu and Kane^42^ to calculate topological invariant 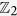. We state a time-reversal invariant periodic Hamiltonian 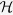 with 2*N* occupied bands characterized by Bloch wave functions. A time-reversal operator matrix relates time-reversed wave functions is defined by


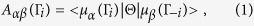


where *α*, *β* = 1, 2, …, *N*, |*μ*_*α*_(Γ_*i*_)> are cell periodic eigenstates of the Bloch Hamiltonian, Θ = exp(*iπS*_*y*_)*K* is the time-reversal operator (*S*_*y*_ is spin and *K* complex conjugation), which Θ^2^ = −1 for spin 1/2 particles. Since 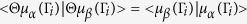, 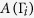 is antisymmetric at TRIM Γ_*i*_. The square of its Pfaffian is equal to its determinant, i.e., det[*A*] = Pf[*A*]^2^. Then *δ*_*I*_ = (det[*A*(Γ_*i*_)])^1/2^/Pf[*A*(Γ_*i*_)] = ±1. Hence, the topological invariant 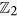 can be defined as


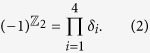


When solids have space-reversal symmetry, 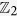 can be simplified as


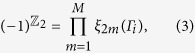


where *ξ* is the parities of all occupied bands at Γ_*i*_, and *M* is the number of Kramers pairs.

### Electronic structure calculation

We calculate the lattice configurations as well as electronic band structures of bilayer phosphorene with four different stacking orders based on the density functional theory (DFT) implemented in the Vienna Ab-initio Simulation Package (VASP)^51^,^52^. The projector augmented wave (PAW)^53^ method and the Perdew-Burk-Ernzerhof (PBE)^38^ exchange-correlation functional are adopted. Long-range dispersion corrections have been taken into account within a semi-empirical van der Waals approach proposed by Grimme known as the DFT-D2 method^43^ (where D2 stands for the second generation of this method). The kinetic energy cutoff for the plane wave basis set is chosen to be 600 eV, and the reciprocal space is meshed at 14 × 10 × 1 using Monkhorst-Pack method^54^. A vacuum space of at least 25 Å along the *z* direction is used to separate the bilayer systems in order to avoid spurious interactions due to the nonlocal nature of the correlation energy^55^. In order to correct the PBE bulk bandgaps, we apply a hybrid Heyd-Scuseria-Emzerhof (HSE)^39^,^40^ functional in which the exchange potential is separated into a long-range and a short-range part, where 1/4 of the PBE exchange is replaced by the Hartree-Fock exact exchange and the full PBE correlation energy is added. Hence the HSE functional corrects the GGA bandgaps^40^ significant by partially correcting the self-interaction. Spin-orbit coupling (SOC) is included in the calculations after the structural relaxations^56^.

### Optical response

We calculate the optical responses of the Tb-stacked bilayer phosphorene under the strains *σ*_*xy*_ = 0.0 and −3.0%, based on a real-space and real-time time-dependent density functional theory (TDDFT) as implemented in the OCTOPUS code^57^. The Hartwigsen-Goedecker-Hutter pseudopotentials^58^ and Generalized Gradient Approximation (GGA) with PBE functional for the exchange-correlation are used to calculate both the ground state and excited state. We consider a 4 × 4 supercell of Tb-stacked phosphorene [see Fig. 7(a)] in order to ignore the interaction between the two boundaries. The simulation zone is defined by assigning a sphere around each atom with a radius of 6 Å and a uniform mesh grid of 0.3 Å. The induced density plane paralleled to the bilayer phosphorene plane, and located at the middle of the top pucker-layer in the vertical direction. In the real time propagation, excitation spectrum is extracted by Fourier transform of the dipole strength induced by an impulse excitation^59^. In the real-time propagation, the electronic wave packets are evolved for typically 6000 steps with a time step of Δ*t* = 0.005 Å/eV. The obtained optical bandgap is close to the electronic bandgap calculated by PBE implemented in VASP, which means that OCTOPUS is reliable on calculation for the optical property.

## Additional Information

**How to cite this article**: Zhang, T. *et al.* Stacked bilayer phosphorene: strain-induced quantum spin Hall state and optical measurement. *Sci. Rep.* **5**, 13927; doi: 10.1038/srep13927 (2015).

## Supplementary Material

Supplementary Information

## Figures and Tables

**Table 1:** The structural parameters of the four different stacking orders obtained by using PBE functional, as compared with that by using HSE06 founctional^26^ and the experimental data for bulk black phosphorus^60^, where *a* and *b* are the lattice constants along x and y directions, respectively, *d*_*int*_ is the smallest interlayer distance, *R*_1_ and 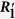 are the P-P bond lengths in up and bottom sublayers of a nonplanar monolayer, respectively, *R*_2_ is the P-P bond length between up and bottom sublayers of a nonplanar monolayer, *α* is the angle between two *R*_1_ bonds, *β* is the angle between the *R*_2_ and 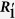 bonds, *E*_*coh*_ is the cohesive energy, and Δ*E*_*coh*_ is the relative difference of the cohesive energy for Ta, Tc, and Td stacking orders and the Tb stacking order.

order		*a*	*b*	*d*_*int*_	*R*_1_	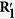	*R*2	*α*	*β*	*Ecoh*	Δ*Ecoh*
		(Å)						(°)		(eV/atom)	
(Ta)	PBE	3.314	4.519	3.503	2.221	2.226	2.256	96.45	103.26	−3.6550	0.008
	HSE06^26^	3.326	4.550	3.495	2.243	2.235	2.283				
(Tb)	PBE	3.319	4.505	3.108	2.223	2.226	2.253	96.55	103.17	−3.6630	0.0
	HSE06^26^	3.331	4.526	3.214	2.242	2.238	2.277				
(Tc)	PBE	3.312	4.546	3.739	2.223	2.224	2.251	96.34	103.54	−3.6556	0.0074
	HSE06^26^	3.324	4.535	3.729	2.238	2.236	2.274				
(Td)	PBE	3.315	4.524	3.291	2.221	2.225	2.255	96.50	103.34	−3.6559	0.0071
	Exp^60^	3.314	4.376	3.503	2.224		2.244	96.34	102.09		

**Table 2:** Products of parity eigenvalues under *σ*_*xy*_ = 0.0 and −3.0% at four time-reversal invariant momenta, i.e., Γ, X, Y, and M. Even (odd) parity is denoted by + (−).

Strain	Γ	X	Y	M	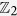
0.0	+	+	+	+	0
−3.0%	−	+	+	+	1

The resulting *Z*_2_ values are given.
