# Supplementary material for: Stacked bilayer phosphorene: strain-induced quantum spin Hall state and optical measurement
Source: Sci Rep. 2015 Sep 15;5:13927. doi: 10.1038/srep13927 (PMC4570210; doi:10.1038/srep13927)
Supplement: Supplementary Information [file srep13927-s1.pdf]

Supplementary information for "Stacked bilayer phosphorene: strain-induced quantum spin Hall state and optical measurement"

Tian Zhang[1,2], Jia-He Lin[1,2], Yan-Mei Yu[1⋆], Xiang-Rong Chen[2⋆] and Wu-Ming Liu[1⋆]

[1]*Beijing National Laboratory for Condensed Matter Physics, Institute of Physics, Chinese Academy of Sciences, Beijing 100190, China*

[2]*Institute of Atomic and Molecular Physics, College of Physical Science and Technology, Key Laboratory of High Energy Density Physics and Technology of Ministry of Education, Sichuan University, Chengdu 610065, China*

⋆*e-mail:ymyu@iphy.ac.cn;xrchen@scu.edu.cn;wliu@iphy.ac.cn*

Structural stability of bilayer phosphorene for Tb stacking order. The structural stability of the Tb-stacked bilayer phosphorene under the high compression strain σ_{xy} can be checked by studying the phonon spectrum (see Fig. 1S). The phonon dispersion of the Tb-stacked bilayer phosphorene have 24 branches because its unit cell contains 8 atoms, where 3 branches are acoustic and the remaining 21 branches are optical branches. The phonon modes under σ_{xy}=0 are doubly degenerate above 10 THz due to weak coupling between the top and bottom layers. While the double degeneracy becomes to lift under high compression strain, such as σ_{xy}=−3.0% and σ_{xy}=−5.0%. The phonon spectrum without imaginary frequency shows that the Tb-stacked bilayer phosphorene is stable when σ_{xy}=0.0, −3.0% and −5.0%.

Optical property of bilayer phosphorene for Tb-stacked order Fig. 2S presents the Fourier transform of the induced charge density in the normal state under σ_{xy}=0 and the quantum spin Hall (QSH) state under σ_{xy}=−3.0% for incident light polarized along the zigzag (x) and armchair (y) directions. When the incident light is polarized in the zigzag direction, the induced electron and hole densities are separate, locating at the left and right sides, respectively [as denoted by the black and white boxs in Fig. 2S(a) and (c)]. Furthermore, we can find that the induced charge density in the QSH state is lower than the normal state by comparing Fig. 2S(a) and (c). This explains why the absorption spectrum in the QSH state is weaker than the normal state. When the incident light is polarized along the armchair direction, as shown in Fig. 2S(b) and (d), the induced electron and hole densities are distributed at the up and down sides (as denoted by the black and white boxs), respectively. Furthermore, the induced charge density in the QSH state is richer and concentrated in the center of the bilayer phosphorene when incident light is polarized along the armchair direction. This is contributed to that the shielding effect becomes stronger in QSH state when the interatomic spacing becomes smaller due to the in-plane strain.

Figure 1S Phonon spectra of bilayer phosphorene for Tb stacking order under different σ_{xy} values. (a), (b) and (c) The phonon spectra when σ_{xy} = 0.0, −3.0% and −5.0%, respectively. The red rectangle in (a) shows the first Brillouin zone, where Γ, S, X and Y refer to special points in the first Brillouin zone. The insets of (a), (b) and (c) show the phonon dispersion around Γ.

Figure 2S Induced charge density of bilayer phosphorene for Tb stacking order under different σ_{xy} values by PBE method. (a) and (b) The induced charge density to an impulse excitation polarized in the *x* and *y* directions when σ_{xy}=0. (c) and (d) The induced charge density to an impulse excitation polarized in the *x*- and *y*-directions when σ_{xy}=−3.0%. The regions where electron and hole density mainly locates are highlighted by black and white dashed boxes. The selected energy resonance points are 4.96 eV (a), 1.11 eV (b), 4.96 eV (c) and 1.22 eV (d).

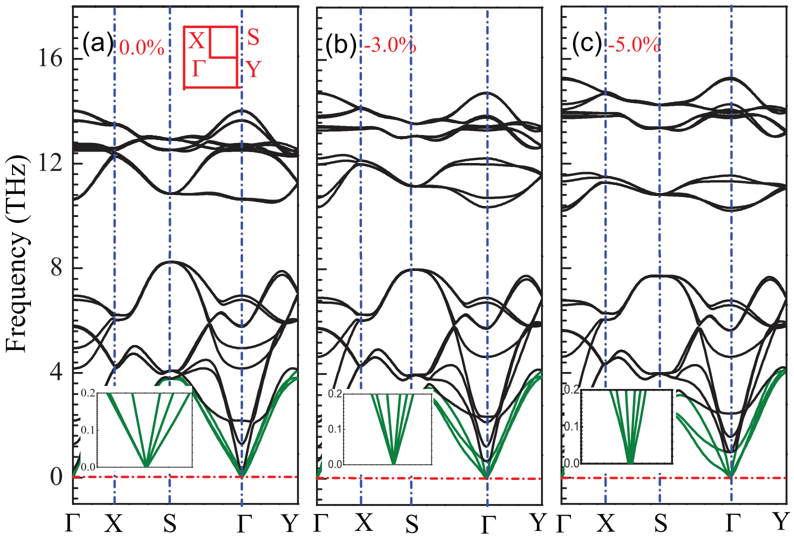

Frequency (THz)
(a)
0.0%
(b)
-3.0%
(c)
-5.0%
X
S
Γ
Y
0
4
8
12
16
0.0
0.1
0.2
Γ X S Γ Y

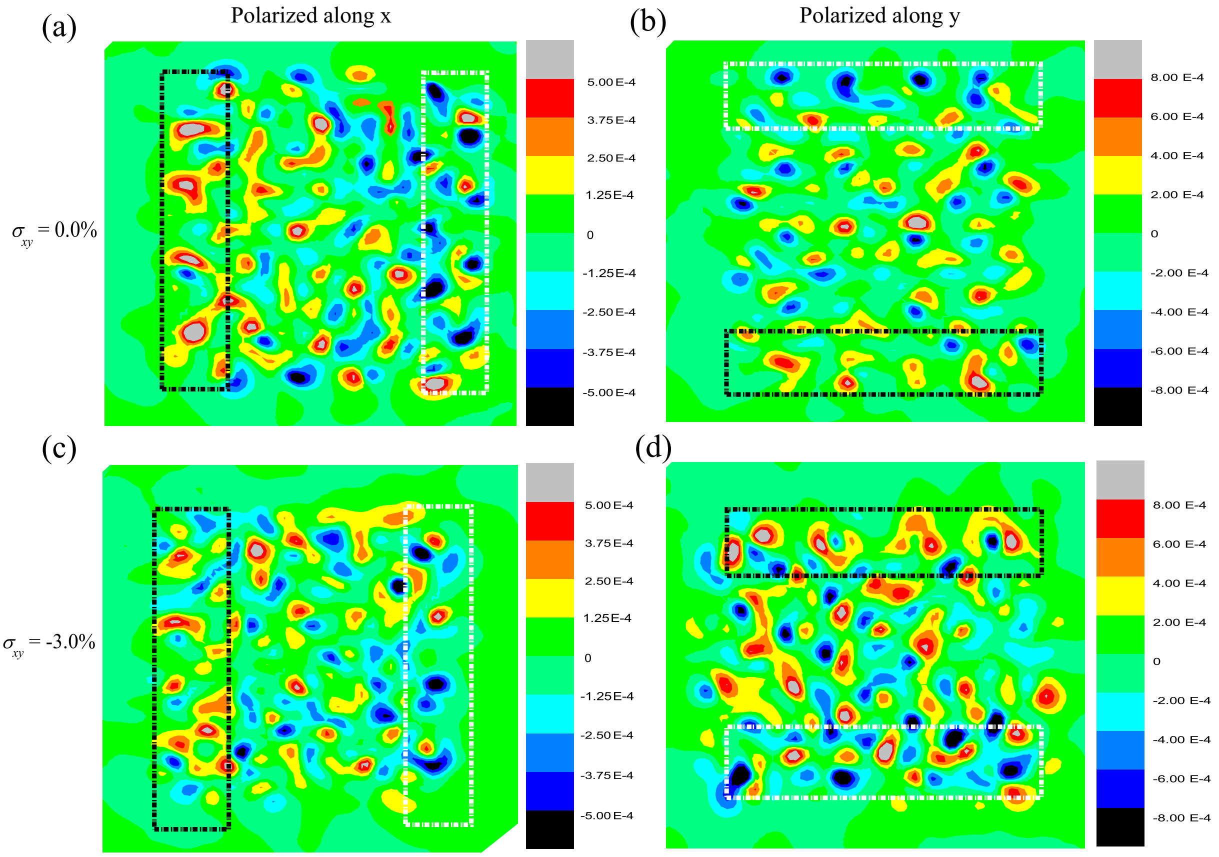
(a)
Polarized along x
(b)
Polarized along y
σ_{xy} = 0.0%
5.00E-4
3.75E-4
2.50E-4
1.25E-4
0
-1.25E-4
-2.50E-4
-3.75E-4
-5.00E-4
8.00 E-4
6.00 E-4
4.00 E-4
2.00 E-4
0
-2.00 E-4
-4.00 E-4
-6.00 E-4
-8.00 E-4
(c)
(d)
σ_{xy} = -3.0%
